# The effects of electrochemical pretreatment and curing environment on strength and leaching of stabilized/solidified contaminated sediment

**DOI:** 10.1007/s11356-023-31477-6

**Published:** 2023-12-22

**Authors:** Anna Norén, Ann-Margret Strömvall, Sebastien Rauch, Yvonne Andersson-Sköld, Oskar Modin, Karin Karlfeldt Fedje

**Affiliations:** 1 Division of Water Environment Technology, Department of Architecture and Civil Engineering, Chalmers University of Technology, 412 96 Gothenburg, Sweden; 2 Swedish National Road and Transport Research Institute (VTI), Box 8072, 402 78 Gothenburg, Sweden; 3 Division of Geology and Geotechnics, Department of Architecture and Civil Engineering, Chalmers University of Technology, 412 96 Gothenburg, Sweden; 4 Recycling and Waste Management, Renova AB, Box 156, 401 22 Gothenburg, Sweden

**Keywords:** TBT, Zn, Leaching, Compressive strength, Curing, Stabilization and solidification, Salinity, Dissolved organic carbon

## Abstract

Stabilization and solidification (S/S) is known to improve the structural properties of sediment and reduce contaminant mobility, enabling the utilization of dredged contaminated sediment. Further reduction of contaminants (e.g., tributyltin (TBT) and metals) can be done using electrochemical treatment prior to S/S and could potentially minimize contaminant leaching. This is the first study on how electrochemical pretreatment affects the strength and leaching properties of stabilized sediments. It also investigates how salinity and organic carbon in the curing liquid affect the stabilized sediment.

The results showed that the electrolysis reduced the content of TBT by 22% and zinc by 44% in the sediment. The electrolyzed stabilized samples met the requirements for compression strength and had a reduced surface leaching of zinc. Curing in saline water was beneficial for strength development and reduced the leaching of TBT compared to curing in fresh water. The results indicate that pretreatment prior to stabilization could be beneficial in reducing contaminant leaching and recovering metals from the sediment. The conclusion is that a better understanding of the changes in the sediment caused by electrochemical treatment and how these changes interact with stabilization reactions is needed. In addition, it is recommended to investigate the strength and leaching behavior in environments similar to the intended in situ conditions.

**Supplementary Information:**

The online version contains supplementary material available at 10.1007/s11356-023-31477-6.

## Introduction

Regular dredging is needed to maintain sufficient water depth in ports. Just in the European Union (EU), ~ 90 million tonnes of sediments were dredged in 2018 (Eurostat, n.d.). As the dredged masses are often contaminated, there is a need to develop treatment techniques and management strategies to sustainably deal with the sediments. Contaminants typically include hazardous organotin compounds (e.g., tributyltin) and metals. Tributyltin (TBT) is a highly toxic man-made product with reported negative impacts on biota at concentrations as low as 0.2 ng/L (European Commision, 2005). It was used in boat paint to prevent fouling on ship and leisure boat hulls, but due to its toxic properties, its usage in paint was banned for small boats in 1989 and larger vessels in 2003 in the EU (EU Regulation (EC) No. 782/2003 and Directive 89/677/EEC). The degradation of TBT in sediments is slow, and high TBT content is still to be found despite the prohibitions. In aerobic and light conditions, the half-life is 1 to 3 years, while in dark and anaerobic conditions, the half-life is expected to be around 10 to 90 years (Dowson, et al. 1996; Viglino, et al. 2004). The degradation of TBT occurs in multiple steps through debutylization. Tributyltin degrades into dibutyltin (DBT), then monobutyltin (MBT), and finally inorganic Sn. In general, the toxicity is reduced by each degradation step (Antizar-Ladislao 2008). Leaching of metals from the sediments is also a concern as elevated concentrations of some metals, e.g., Cu and Zn, could harm biota (Besser, et al. 2018; Jakimska, et al. 2011). The level of contamination and existing legislation are determining factors for the choice of management option (Casper 2008; Norén, et al. 2020). Internationally, the most common sediment management methods are disposal at a landfill or in the sea (Akcil, et al. 2015; Bortone, et al. 2004). In the EU, legislation changes imposed more restrictions on landfilling materials such as soil and sediment, and as a consequence, landfilling costs are increasing (European Environment Agency, 2009). This encourages the development of techniques that allow sediment reuse. An increasingly used technique is stabilization and solidification (S/S), in which dredged material is mixed with binders, such as, e.g., cement and ground granulated blast-furnace slag (GGBS). Since the production of cement releases large quantities of CO_2_ (Kim, et al., 2017), GGBS could be used to replace a part of the cement to reduce the climate impact (Zhang, et al. 2020). In addition, GGBS also has a positive impact on strength development in the stabilized sediment. In the S/S technique, the sediment is hardened (i.e., solidifies), which improves its structural strength and could enable its use, e.g., in port constructions. The technique does not only solidify the masses, but it also reduces the permeability and prevents the spread of some contaminants (i.e., stabilizes). To further reduce the risk of contaminant leaching, pretreatment could potentially be used to reduce the contaminant content before S/S is done. A pretreatment capable of reducing the sediment’s TBT and metal content is electrochemical oxidation (Norén, et al. 2022). Additionally, metals could potentially be recovered during this treatment. However, electrochemical pretreatment may affect the sediment and impact the stabilized sediment’s strength development and leaching. The release of elements from building materials is usually determined using standardized leaching tests (e.g., EA-NEN7375:2004 and SS-EN 12457–4 tests), but in these, water similar to the field condition is typically not used. Marine-stabilized sediments would, however, typically be used in marine environments, surrounded by saline water containing dissolved organic carbon (DOC). The salinity of leaching agents has been seen to impact the leaching of metals and TBT from sediment (Norén, et al. 2021). Thus, standardized leaching tests using demineralized water as a leaching agent might give an incorrect estimate of the leaching in field conditions.

The aim of this study was to investigate how electrochemical pretreatment degrades TBT and removes metals and influences the strength and leaching properties once the sediments are stabilized. Untreated and electrochemically pretreated sediments were stabilized with GGBS and cement, and the surface leaching and mechanical properties of the stabilized material were studied over time. Diffusion rates and leaching mechanisms in water with varying salinity and DOC concentration were investigated and compared, to identify how leaching differs between standardized test conditions and field conditions. Additionally, the impact of the surrounding water salinity during the curing on strength development was investigated.

To the best of our knowledge, this is the first study of how electrochemical pretreatment affects the leaching and compressive strength of stabilized sediment. The results of this study will be useful for stakeholders involved in sediment management, enable the use of treated sediment, and motivate further research in this area.

## Materials and methods

**Fig. 1:**
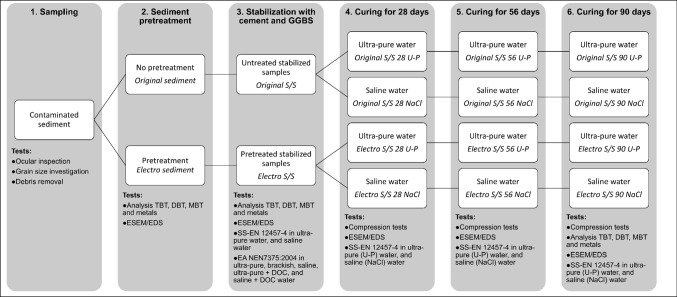
Overview of the testing procedure, steps 1 to 6. Sample names used are marked in italics in each box. The number in sample names denotes the age of the stabilized and solidified (S/S) test piece, U-P denotes samples being cured in ultra-pure water, and NaCl denotes samples being cured in saline conditions. Electro denotes electrochemically pretreated samples, while original denotes untreated samples

An overview of the testing procedure step by step is presented in Fig. 1.

### Sampling and electrochemical pretreatment (steps 1–2)

Sediment was collected during a dredging operation in the estuary of the river Göta älv in Gothenburg, Sweden. In total, 40 containers of 5 L each were collected. Ocular inspection of the sediment was done, and coarser objects (e.g., mussel shells) were removed. The collected sediment was frozen to − 22 °C and thawed at room temperature before use. Metal and organotin analysis and environmental scanning electron microscopy imaging showed that freezing and thawing did not cause changes in element concentration or sediment composition. Before stabilization, some of the sediment was electrochemically pretreated according to a procedure adapted from Norén et al. (2022). The sediment was treated in a 1-L glass beaker in batches of 0.5 kg (~ 40% dry weight) at 23 V for 24 h with an average of 0.77 A. The electrodes used had dimensions of 10 × 10 cm and were placed 9 cm apart. A boron doped diamond-coated niobium plate (Nb/BDD) (Neocoat, Switzerland) was used as an anode, and a titanium plate (Alfa Aesar) was used as a cathode.

### Stabilization with cement and GGBS (step 3)

Both untreated (original) and electrolyzed (electro) sediments were stabilized to investigate how the pretreatment affects strength development and leaching of organotin compounds and metals. Both types of sediment were mixed with GGBS (Ecocem, n.d.) (Appendix Table A.1) and cement of type CEM II/A-LL 42.5R (Byggcement Std PK Skövde, Cementa (Cementa 2021)) (Appendix Table A.2) according to the recipe used for the construction of a new port terminal in Gothenburg, Sweden (Göteborgs hamn 2019). The recipe was developed specifically for the original sediment from this site, and wet sediment was used without any water added, resulting in a water-binder ratio of ~ 4 for all samples.

**Table 1:** Total organic content (TOC), tributyltin (TBT), dibutyltin (DBT), monobutyltin (MBT), and selected metals content in untreated (original) and electrochemically pretreated (electro) sediment

	Original sediment	Electro sediment
TOC (% DW)	2.05	2.43
Organotin compounds (µg/kg DW)		
TBT	170	130
DBT	40	29
MBT	21	21
Metals (mg/kg DW)		
Cd	0.33	0.24
Cu	35	29
Zn	130	72

**Table 2:** Total organic content (TOC), tributyltin (TBT), dibutyltin (DBT), and monobutyltin (MBT) content in untreated (original) and electrochemically pretreated (electro) stabilized samples (S/S). Sample names including 90 U-P and 90 NaCl refer to stabilized samples cured for 90 days in ultra-pure water (U-P) and saline (NaCl) water, respectively

	Original S/S	Electro S/S	Original S/S 90 U-P	Original S/S 90 NaCl	Electro S/S 90 U-P	Electro S/S 90 NaCl
TOC (% DW)	3.6	2.8	2	2.1	2	1.9
Organotin compounds (µg/kg DW)						
TBT	82 ± 7.1	75 ± 21	32 ± 6.6	98 ± 8.8	35 ± 22	37 ± 16
DBT	14 ± 0.14	14 ± 8.8	5.1 ± 0.74	13 ± 4.3	9 ± 0.71	8.3 ± 1.2
MBT	22 ± 1.7	24 ± 1.2	7.1 ± 4.5	17 ± 1.2	10 ± 10	12 ± 5.5

Sediment and binders were mixed using a cement mixing rod for 5 min to get a homogeneous binder content of 75 kg/m^3^ cement and 75 kg/m^3^ GGBS. The stabilized sediment was cast in 100 × 100 × 100 mm molds and 40 × 40 × 160 mm molds to be used in compression tests and leaching tests, respectively. The molds were covered with plastic foil, and after 24 h, the stabilized samples were removed from the molds. The surface diffusion test (EA NEN7375:2004) was started directly after the 40 × 40 × 160 mm test pieces were removed from the molds, while the 100 × 100 × 100 mm test pieces were submerged in ultra-pure (Thermo Scientific, 18.2 MW cm) or saline (35 g/L NaCl) water during the curing (28, 56, and 90 days) and were used for compression tests and thereafter the leaching test for crushed material (SS-EN 12457–4).

The surface diffusion test (EA NEN7375:2004 (NEN7375)) was done to evaluate leaching characteristics from the stabilized sediment over time (Environment Agency, 2005). At predefined intervals (0.25, 1, 2.25, 4, 9, 16, 36, and 64 days), the leaching agent was changed and analyzed. By plotting the cumulative leaching (e^*^_n_) and derived leaching (e_n_) curves over time leaching mechanisms such as surface wash-off, diffusion, and depletion could be identified by studying the slope of the leaching curves. A curve with a slope > 0.35 and ≤ 0.65 (ideally 0.5) indicates that diffusion is occurring, while a slope > 0.65 could be due to dissolution or delayed diffusion, and a slope ≤ 0.35 could indicate that surface wash-off or depletion is occurring (Environment Agency, 2005). To investigate how water characteristics impact the surface leaching of the stabilized samples, water with varying salinity and organic content was used (ultra-pure water, brackish water (15 g/L NaCl), saline water (35 g/L NaCl), and ultra-pure and saline water with added DOC (6 mg/L humic acids was also used, Aldrich, prepared according to Florence (1982))). One-day-old stabilized samples were used in this leaching test.

### Curing for 28, 56, and 90 days (steps 4–6)

An overview of the performed tests and sample names is presented in Fig. 1 Compressive strength tests were performed on days 28, 56, and 90 on original and electro stabilized sediments that had been cured in ultra-pure or saline water. An MTS 880 servohydraulic testing machine was used to compress the samples. The loading rate was 1 mm/min, and all tests were performed in either triplicates or quadruplicates. Each specimen was weighed and measured before the test

The compliance test for leaching of granular waste material (SS-EN 12457–4 (L/S10)) was done on stabilized sediment to investigate the risk of leaching in a worst-case scenario, i.e., the material is crushed or eroded (Swedish Standards Institute, 2003). The test was done using standard ultra-pure water and saline water (35 g/L NaCl) on original and electro stabilized samples after curing for 1, 28, 56, and 90 days. Before the leaching test, the stabilized sediments were manually grounded down to a particle size of < 10 mm using a mortar and pestle.

### Chemical and physical analysis

After sampling, the sediment’s grain size distribution was determined according to ISO 11277:2009 (International Organization for Standardization, 2009). The sediment’s loss on ignition (LOI) and dry matter (DW) were measured using the method SS-EN 028113 (Swedish Standard Institute, 1981). Total organic content (TOC) was measured according to the methods CSN ISO 10694:1995 (International Organization for Standardization, 1995), CSN EN 13137:2002 (European Standard, 2001), and CSN EN 15936:2012 (European Standard, 2012). The organotin compounds TBT, dibutyltin (DBT), and monobutyltin (MBT) were analyzed in sediment and stabilized sediment at an external accredited laboratory using SS-EN ISO 23161:2011 (International Organization for Standardization, 2011) and using ISO17353:2004 (International Organization for Standardization, 2004) for leachates. The standards SS-EN ISO 17294–2:2016 (Swedish Standard Institute, 2016a, 2016b) and US EPA method 200.8:1994 were used for total amount analyses of major and minor elements (Ag, As, Ba, Cd, Co, Cr, Cu, Hg, Mo, Ni, Pb, Sb, Sn, V, and Zn) in sediment. Major and minor elements in leachates were analyzed by ICP-MS using a Thermo Scientific ICAP Q instrument with an SC-FAST sample introduction system. Each leachate sample was divided, where one part was filtered with a 0.45-µm filter, while the other part was not filtered to investigate if the elements were attached to suspended particles or dissolved. The samples were then diluted to levels in the instrument’s analytical range and acidified with nitric acid.

The morphology, shape of particles, and investigation of element distribution in original sediment, stabilized sediment (days 3, 29, 57, and 90), cement, and GGBS were investigated using an environmental scanning electron microscopy (ESEM, FEI Quanta 200 FEG-ESEM) with an Oxford INCA energy dispersive X-ray spectrometer (EDS). The samples were mounted on carbon tape and dried at ambient conditions without any coating procedure. Low vacuum pressure was used during the analysis. See Fig. 1 for an overview of the analysis performed in the different steps.

## Results and discussion

### Characterization of original, electrochemically pretreated, and stabilized sediments

Grain size analysis showed that the original sediment consisted mainly of silt (41%), sand (39%), and clay (21%). The electrochemical pretreatment had no or only limited effect on sediment particle size (Appendix Fig. A.1 (Fig. A.1)). These results indicate that the sediment after electrolysis is expected to have approximately the same properties as the untreated original sediment in the stabilization experiments.

#### Comparison of contaminant content in original and electrochemically pretreated sediment

The electrochemically treated (electro) sediment contained about 20% less TBT than the original sediment (Table 1). This was a lower TBT reduction than earlier reported by Norén et al. (2022), where TBT was reduced by 58%. One explanation for the difference in removal rate is that larger batches were treated in this study (0.5 kg instead of 0.2 kg wet sediment) and that the equipment used was not scaled up for this experiment. The reason for treating a larger sediment quantity was to produce a sufficient amount of sediment for stabilization within the project’s time frame. A better adjustment of the experiment equipment could potentially further reduce the TBT content. Additionally, the treatment efficiency could have decreased over time due to anode delamination as large quantities of sediment were treated to produce a sufficient amount needed for the stabilization (Lu, et al. 2019; Norén, et al. 2021). Both original and electro sediments were classified to have *very high TBT content*, according to the Swedish sediment classification system (Josefsson 2017) and causing *extensive acute toxic effects* according to the Norwegian classification (Direktoratsgruppen vanndirektivet 2018). The derivate DBT was reduced by nearly 30%, but MBT content was similar for electro and original sediments (Table 1); both concentrations were considered to be in *high content* according to the Swedish sediment classification system (Josefsson 2017).

For all the investigated metals, only Cd, Cu, and Zn exceeded differential environmental guidelines for sediment. The original sediment’s metals Cd, Cu, and Zn were above the Norwegian background levels but do not cause toxic effects (Direktoratsgruppen vanndirektivet 2018). The Zn content had a large deviation from preindustrial metal content in sediment (class IV) in the original sediment (Table 1), and the Cu content distinctly deviates from the same preindustrial content (class III) (Naturvårdsverket 1999). Copper and Zn in the original sediment are also exceeding the interim sediment quality guidelines but are below the probable effect levels according to the Canadian classification sediment (Canadian Council of Ministers of the Environment, n.d.).

After the electrochemical treatment, the sediment had lower metal contents and a reduced content-based environmental risk (Norén, et al. 2022). The most significant reduction was for Zn with a removal of 44% (Table 1). This reduced the Zn in the electro sediment to levels below the Swedish preindustrial levels (class I), Canadian interim sediment quality guideline, and Norwegian background levels (Canadian Council of Ministers of the Environment, n.d.), Direktoratsgruppen vanndirektivet 2018; Naturvårdsverket 1999). The electrolysis additionally reduced the Cu content to class II (small deviation from preindustrial levels) (Naturvårdsverket 1999).

#### Comparison of contaminant content in stabilized sediments

After stabilization, the TBT content was reduced in both original and electro stabilized sediment (Table 2). Measured TBT contents in the stabilized specimens after crushing were 82 and 75 µg/kg DW for original and electro stabilized sediments, respectively. This is 46% less than in the original sediment and 37% less than in the electro sediment than what it theoretically would contain after the dilution by the addition of GGBS and cement (Tables 1 and 2). This difference could be due to the alkaline pH and the stabilizing effect preventing TBT from being extracted during chemical analysis as the method is designed for soil-like materials (International Organization for Standardization, 2011). Analyses of the stabilized original and electro stabilized sediment specimens after 90 days of curing in ultra-pure or saline water and after crushing showed a further reduction in the content or availability of TBT. This could be due to an improved stabilizing effect, potential degradation within the samples, or leaching during the curing. The difference in TBT content between the electro and original stabilized sediment decreased with time. However, the sample Original S/S 90 NaCl (Table 2) is an exception where the TBT content seems to remain unaffected over time. Ultra-pure water was previously reported to reduce the TBT content in sediment by promoting leaching, whereas high salinity seemed to result in a lower release (Norén, et al. 2021). The higher ionic strength may prevent TBT or important stabilizing agents to be leached out.

ESEM images of original and electro sediments show similar grains being present in the sediment, indicating that the electrochemical treatment has not affected the sediment grain size (Fig. A.1). However, some differences appear between the 1-day-old stabilized and solidified (non-cured) samples. In the Electro S/S sample, the formation of longer crystals was more visible than in the Original S/S sample. This could indicate that some of the curing reactions occurred faster in the electro stabilized sediments, which could impact the properties of the stabilized sediment (Tian and Cohen 2000). For example, if some reactions are postponed and expand the previous hardened structure, this would result in a more brittle stabilized sample and a dramatically decreased strength (e.g., the alkali-silica reaction). On day 90, a lot more needle-shaped crystals, presumably ettringite, were seen in sample Original S/S 90 NaCl in comparison to the other 90-day samples. This could indicate a better S/S effect, preventing TBT from being leached out or degrade within the sample, which could explain the higher TBT content in the sample (Table 2).

### Compressive strength test

**Fig. 2:**
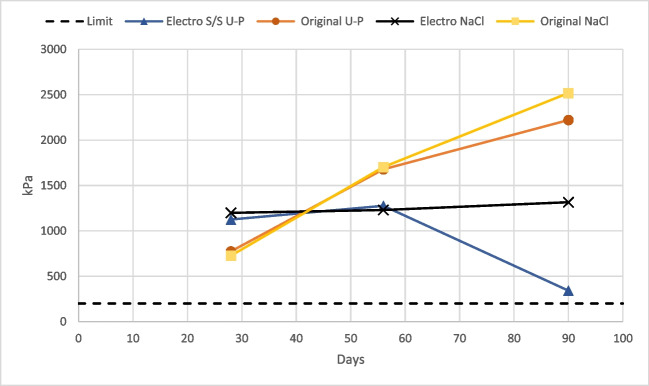
Average compressive strength for electrochemically pretreated stabilized (Electro S/S) and untreated stabilized (Original S/S) samples cured in ultra-pure (U-P) and saline (NaCl) water. The black dashed line corresponds to the compressive strength requirement of 140 kPa (Göteborgs hamn 2019)

The compressive strength of the stabilized sediments varied depending on the sample pretreatment and curing conditions (Fig. 2 and Appendix Table A.3). All tested samples passed the limit set for the shear strength to be used in construction in the Port of Gothenburg, 70 kPa (Göteborgs hamn 2019), which corresponds to compression strength of 140 kPa according to the Tresca criterion. After 90 days, the highest strength was reached by the original stabilized samples cured in saline water (2520 kPa), while the electro stabilized sediment cured in ultra-pure water had the lowest strength (341 kPa). All compression tests initially indicate that the electro stabilized samples are stronger than the original stabilized sediment (Fig. 2). However, on days 56 and 90, the highest strength was reached by the original samples, while the strength of the electrolyzed stabilized sediment had a low increase from day 56 in saline water and even decreased in ultra-pure water.

**Table 3:** Leached-out TBT, DBT, and MBT from electrochemically pretreated (electro) and untreated (original) stabilized samples cured for 90 days in ultra-pure water (U-P) or saline water (NaCl) and leached in saline water in the granular waste compliance L/S10 leaching test

Organotin compounds (ng/kg DM)	Original S/S 90 U-P	Electro S/S 90 U-P	Original S/S 90 NaCl	Electro S/S 90 NaCl
TBT	274	312	309	228
DBT	< 100	< 104	< 97	< 104
MBT	< 100	< 104	< 97	< 104

The results indicate that the ionic strength of the surrounding water during the curing becomes more important as time progresses and that higher ionic strength seems to be beneficial for strength development (Giasuddin, et al. 2013). For the electro stabilized sediment, the surrounding water seems to be particularly important to maintain the strength at day 90, see Fig. 2. The stabilized samples’ curing liquids reached the same conductivity regardless if ultra-pure water or saline water was used. This indicates that more ions have been leached out from the stabilized samples cured in low ionic water. The presence of ions in the surrounding water during curing may prevent important reactive species from leaching out due to concentration equilibrium, resulting in a positive impact on the curing effect. The electrolysis could form new compounds in the sediment and create conditions that could cause the solidifying reactions to deviate from the normal curing process as shown in Fig. 2. Such a deviation in time from when the reactions usually occur could, for example, expand the previous hardened structure resulting in a collapse of the inner structure and a decrease in strength. This is indicated by the ESEM images (Fig. A.1) as previously discussed in the chapter “Comparison of contaminant content in stabilized sediments”. The electrochemical treatment had no apparent impact on the TOC (Table 1), but the treatment could potentially alter the form of the organic materials, e.g., from humic acid to fulvic acid in the sediment (Lu, et al. 2020), which could affect the stabilization. Acidic organic matter can neutralize OH^−^ which could negatively affect pozzolanic reactions (Ma, et al. 2016). Additionally, calcium may react with humic acids to form stable calcium humic acid compounds, which might hinder important reactions for strength development to occur (Chen and Wang 2005; Ma, et al. 2016). However, humic acids could also cause the coagulation of organic substances under the influence of salt, which would have a positive impact on strength development (Du, et al. 2019). Fulvic acids may also react with Ca or with Al and prevent important C-A-H from forming. Gutsalenko et al. (2018) also report a decrease in strength with time due to internal carbonation from the decomposition of organic matter at high pH, which could have affected the hydrolysis products, resulting in a lowered amount of the mineral portlandite (Ca(OH)_2_). To lower the risk for a decrease in strength due to the presence of humic acids and fulvic acids, different admixtures could be used, e.g., calcium sulfate for neutralizing humic acids and aluminum sulfate for fulvic acids.

Analysis of the curing liquids indicated that ~ 19% more SO_4_^2−^ was released from original stabilized samples compared to the electro stabilized samples At a high current, there is a risk for side reactions, such as the formation of S_2_O_8_^2−^ from SO_4_^2−^ (Murugananthan, et al. 2008); it was seen that excess of SO_4_^2−^ was negative for the long-term strength development of stabilized sediment (Xing, et al. 2009). In the original stabilized sediment, the SO_4_^2−^ was present from the start and could react or leach out. During the initial curing, S_2_O_8_^2−^ may still be found in the electro stabilized sediment and with time decompose to SO_4_^2−^. If more SO_4_^2−^ becomes available with time, this may influence the sulfate depletion point and subsequent reactions (Marchon and Flatt 2016). Additions of SO_4_^2−^ could lead to a sulfate attack as it reacts with, e.g., tricalcium aluminate (C_3_A), to form complexes within the solidified sample, such as ettringite or gypsum (Tian and Cohen 2000). The new formations could cause an increased volume and break existing previously hardened structures that are important for strength, causing the stabilized sample to become more brittle. Additionally, the decomposition of S_2_O_8_^2−^ in alkaline water can result in the formation of acid, which would impact the compression strength (Shafiee, et al. 2018).

### Leaching tests on stabilized sediment

The first leaching test, EA NEN7375:2004 (NEN7375), indicates how the surface leaching patterns change over time and could be used to estimate the stabilized sediment’s leaching behavior at the site if used for port construction (Environment Agency, 2005). These tests were done on 1-day-old stabilized samples. The second leaching test, SS-EN 12457–4 (L/S10), was in this case used to investigate extreme leaching conditions (e.g., if the stabilized sediment is exposed to extraordinary weathering, or is crushed) (Swedish Standards Institute, 2003). In Sweden, the L/S10 test is commonly used to categorize waste materials before landfilling. The L/S10 tests were done on 1-, 28-, 56-, and 90-day-old specimens (Fig. 1).

#### Surface leaching according to EA NEN7375:2004

##### Organotin compounds

**Fig. 3:**
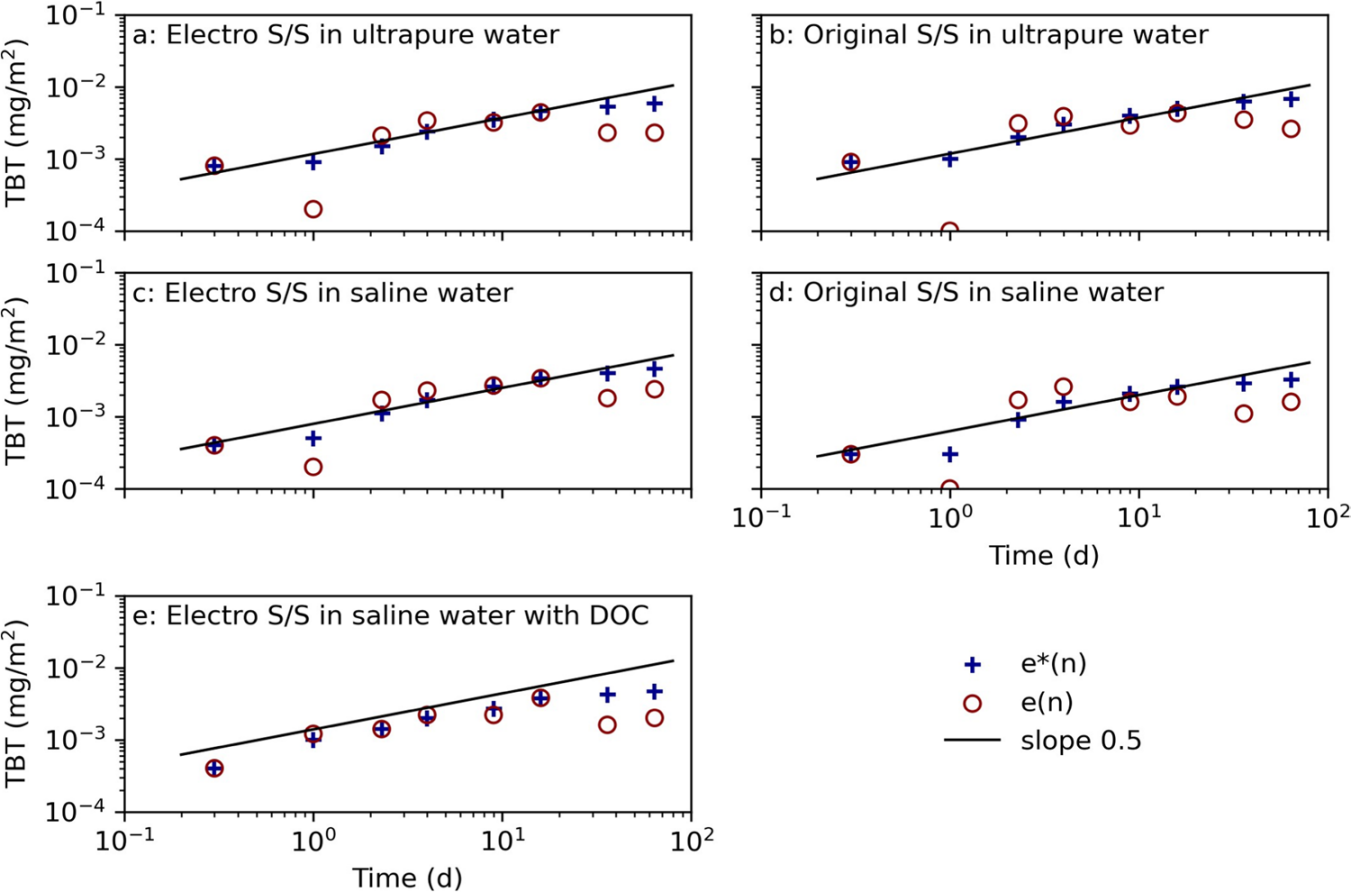
TBT leaching from electrochemically pretreated (electro) and untreated (original) stabilized samples cured in ultra-pure water, saline water, and saline water with dissolved organic carbon (DOC) in the NEN7375 surface diffusion leaching test. The curve e*(n) displays the measured cumulative leaching, and *e*(n) displays the derived cumulative leaching. The slope 0.5 is marked as a reference for identifying diffusion-controlled leaching (Environment Agency (2005))

The TBT leaching curves (Fig. 3) for electro and original stabilized samples show similar TBT leaching curves. The leached-out quantity of TBT after 64 days was higher in ultra-pure water in comparison to saline water. A higher amount of TBT was released in the ultra-pure water, especially in the initial time period (day 0.25), indicating that the stabilizing effect for TBT occurs later in ultra-pure water in comparison to saline water. The ultra-pure water likely extracts more ions from the stabilized samples to reach an ionic equilibrium which may impact the stabilizing effects. The stabilized electrolyzed sediment leached in saline water with DOC displayed a higher TBT release in the initial leaching (day 1) and also leached the most in total in comparison to the other samples. The TBT may bind to the organic parts of the solved humic acid (Fent 1996), as TBT has a higher affinity for organic substances than water and extracted TBT into the leachate. In a study where leaching of TBT from similar sediment was done at varying salinity, pH, DOC concentration, and mixing conditions, it was seen that a saline environment and the presence of humic acid reduced the release of TBT in comparison to leaching in ultra-pure water (Norén, et al. 2021). The higher release in the saline and DOC conditions for the stabilized samples may also be caused by humic acids interfering with S/S reactions on the surficial parts of the samples (Chen and Wang 2005; Ma, et al. 2016), which decreases the stabilizing effects. These results imply that the surrounding salinity impacts the leaching mechanisms of TBT, but other factors, e.g., DOC, are also important.

The slopes of all TBT leaching curves are almost linear to the slope of 0.5, which indicate that TBT is released through surface diffusion from days 2.25 to 16 (Fig. 3) (Environment Agency, 2005). All samples displayed a drop in TBT concentration between days 16 and 36 which may be due to changes in the matrix. This could indicate that readily available TBT is becoming depleted after this time period. During the test longer time intervals (e.g., days 16–36 and 36–64), it is also possible that leached-out TBT starts to degrade into DBT, MBT, and finally Sn. The saline water conditions could enhance the desorption of TBT but also prolong the degradation in comparison to non-saline water (Fent 1996). This could explain why the TBT concentrations in the ultra-pure water leachates start to decline after day 36 but not in the saline leaching conditions.

The leaching curves for DBT (Appendix Fig. A.2 (Fig. A.2)) indicate that the leaching is initially surface diffusion controlled for electrolyzed samples, but after 1 week, dissolution occurs. This is especially noticeable for the electro stabilized sample leached in saline water with DOC by the increasing slope for *e*^n^ (Environment Agency, 2005). However, this change in leaching mechanisms is not seen for the original stabilized sample which seems to be diffusion controlled throughout the experiment. The curves for the derivate compound MBT indicate that leaching is initially diffusion controlled, but around day 9, there is an indication of dissolution starting to occur, especially for the electro stabilized sample in ultra-pure water (Appendix Fig. A.3). The increase in DBT and MBT concentrations in the leachates could indicate TBT degradation is occurring in the water or within the stabilized sample, rather than an increased leaching of the derivates. Dissolved TBT is easier to degrade than TBT attached to solids (Ayanda, et al. 2012; Stewart and de Mora 1990), which means that there is a risk of TBT degrading in the leachate, especially during the longer time intervals at the end of the test. As the test was originally designed for inorganic components (Environment Agency, 2005) and as organotin compounds are degradable, this implies that the results from the leaching of organotin compounds are more difficult to evaluate compared to non-degradable metals. This may result in an underestimation of the TBT leaching and consequently an overestimation of the leaching of DBT and MBT originating from the stabilized samples. Hence, the seen indication of depletion in the graphs for TBT (Fig. 3) in the latter part of the experiment might correlate to the observed increase of DBT during the same time interval (Fig. A.2). The leaching patterns for Sn did not correlate with the leaching of the organotin compounds but instead with other metals, which is explained by that most of the tin is not in the form of organotin compounds.

##### Metals

The S/S reactions are pH dependent before an equilibrium is reached and will also be driven by hydroxide ions if the curing liquid is alkaline (Faraji, et al. 2022). In the NEN7375 leaching tests, the water is exchanged at the given time intervals, and pH measurements showed that the leachates reached a high pH level (~ 11.5) at the end of each time interval. A high pH has an immobilizing effect on most metals, with exceptions like, e.g., the metalloids As and Sb (Johansson, et al., 2009; Wilson, et al. 2010). The pH-controlled leaching is the major driving factor at the beginning of the tests and will be less important during the longer time intervals at the end.

**Fig. 4:**
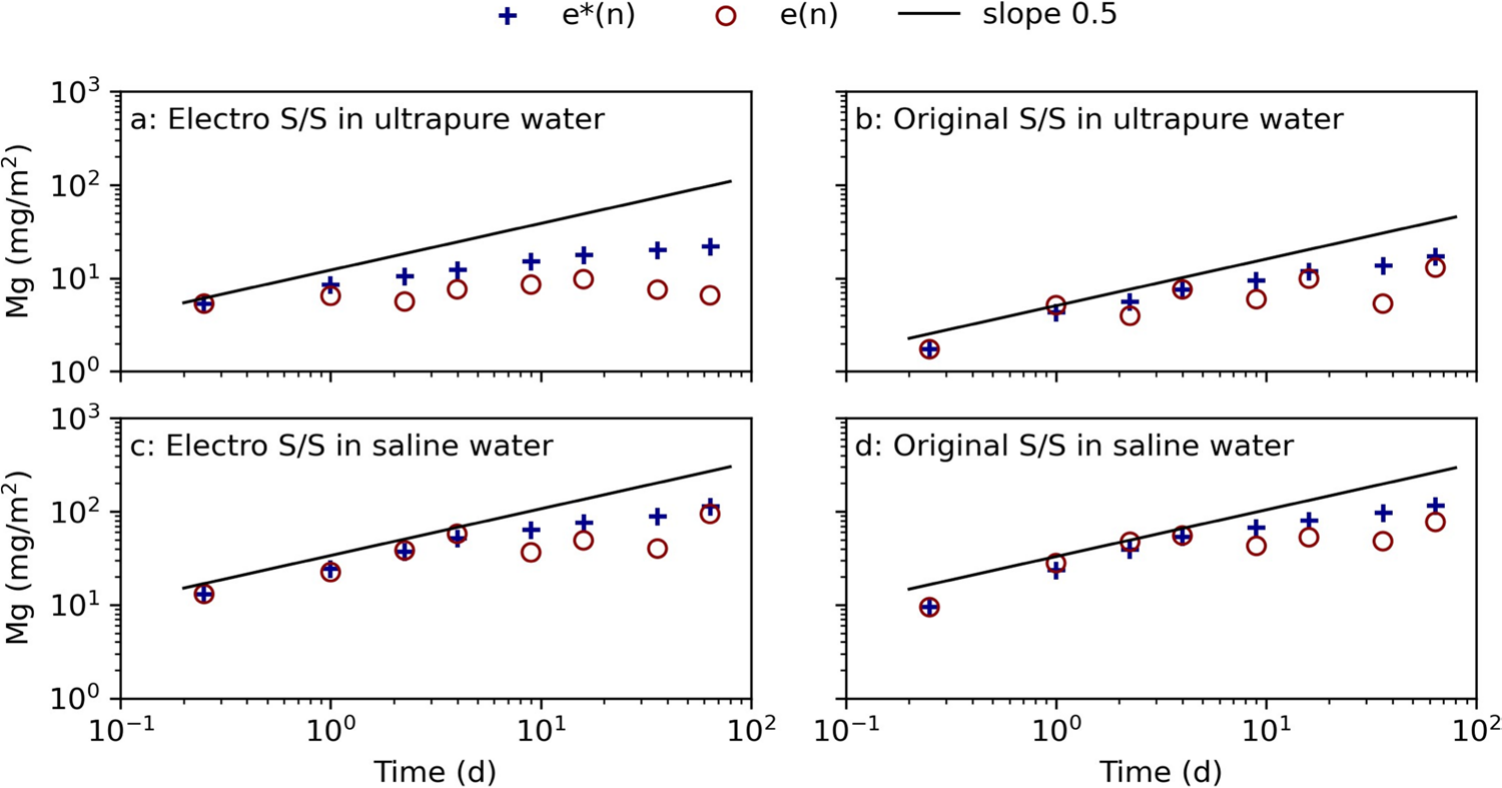
Mg leaching from electrochemically pretreated (electro) and untreated (original) stabilized samples cured in ultra-pure water and saline water in the NEN7375 surface diffusion leaching test. The curve e*(n) displays the measured cumulative leaching, and *e*(n) displays the derived cumulative leaching. The slope 0.5 is marked as a reference for identifying diffusion-controlled leaching (Environment Agency, (2005))

Aluminum, Ca, and Mg are important for the hydrolysis reactions by the formation of, e.g., gibbsite, gypsum, and ettringite (Dijkstra, et al. 2006; Marchon and Flatt 2016). No indication of increased Al mobility was seen in the NEN7375 test (Appendix Fig. A.4) which could have indicated that hydrates have dissolved (Zhang, et al. (2020)). This could be due to the high pH (~ 11.5), at which aluminosilicate minerals rather than gibbsite or ettringite control the Al leaching (Dijkstra, et al. 2006). The leaching curves for Ca for all electro stabilized sediment indicate a possible depletion of different chemical forms, but this is not seen for the original stabilized samples (Appendix Fig. A.5). It is possible that the electrochemical treatment altered the Ca^2+^ binding in the sediment, and later effecting the S/S reactions in the stabilized sediment, as there is a correlation between the high sorption of divalent ions to the stabilized sediment and increased compression strength (Rajasekaran and Narasimha Rao (2002), Xing, et al. (2009)). Dijkstra et al. (2006) showed that the leaching of ettringite follows the leaching trends of Ca at pH between 10 and 12 and that ettringite depleted faster while Ca continued to diffuse over a longer time period. An altered Ca content or formation in the pretreated samples could also trigger a higher formation of Mg-S–H. Analysis of leachate shows that Mg is depleted in the electro stabilized sample in ultra-pure water, which is indicated by the downgoing trend for *e*(n) which is shown in Fig. 4. The same distinct declining leaching curves are not observed for the other samples, where the release mechanisms seem to change less over time. This indicates that the pretreatment itself could have affected the sediment’s physicochemical properties (e.g., cation exchange index) and thereby effect attractive and repulsive forces during the S/S (Sridharan and Rao 1973). The divalent ion Mg^2+^ can replace Ca^2+^ in C-S–H and instead form Mg-S–H, which is a less strong formation that results in a material with lesser strength, as seen for the electro stabilized sediment in Fig. 2.

**Fig. 5:**
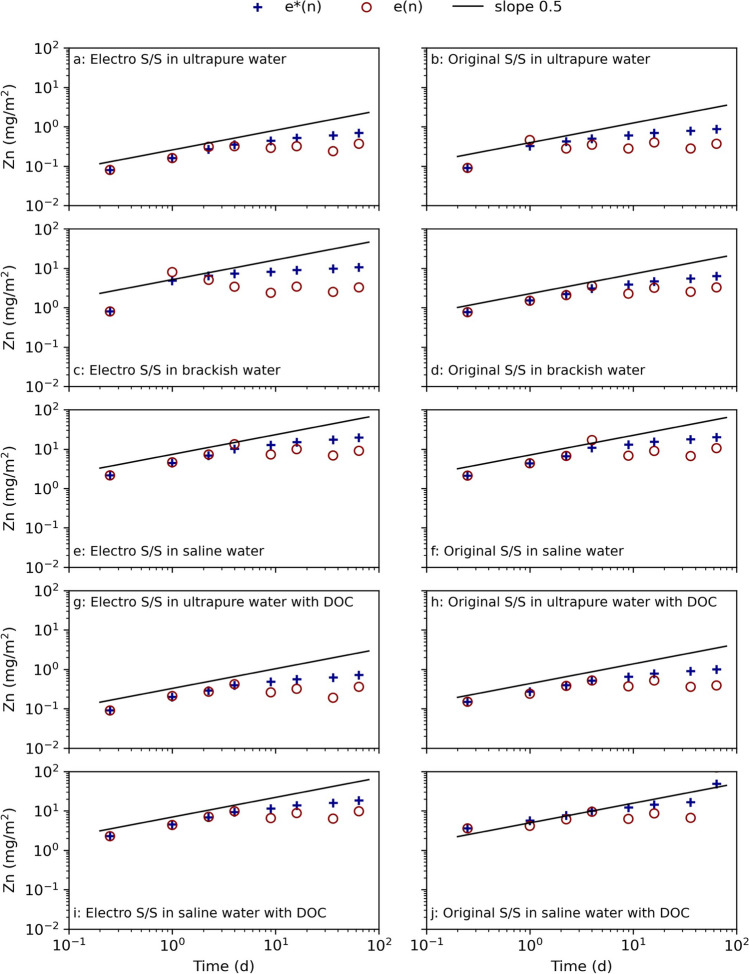
Zn leaching from electrochemically pretreated (electro) and untreated (original) stabilized samples cured in ultra-pure water, brackish water, and saline water, as well as ultra-pure water with dissolved organic carbon (DOC) and saline water with DOC in the NEN7375 surface diffusion leaching test. The curve e*(n) displays the measured cumulative leaching, and *e*(n) displays the derived cumulative leaching. The slope 0.5 is marked as a reference for identifying diffusion-controlled leaching (Environment Agency (2005))

**Fig. 6:**
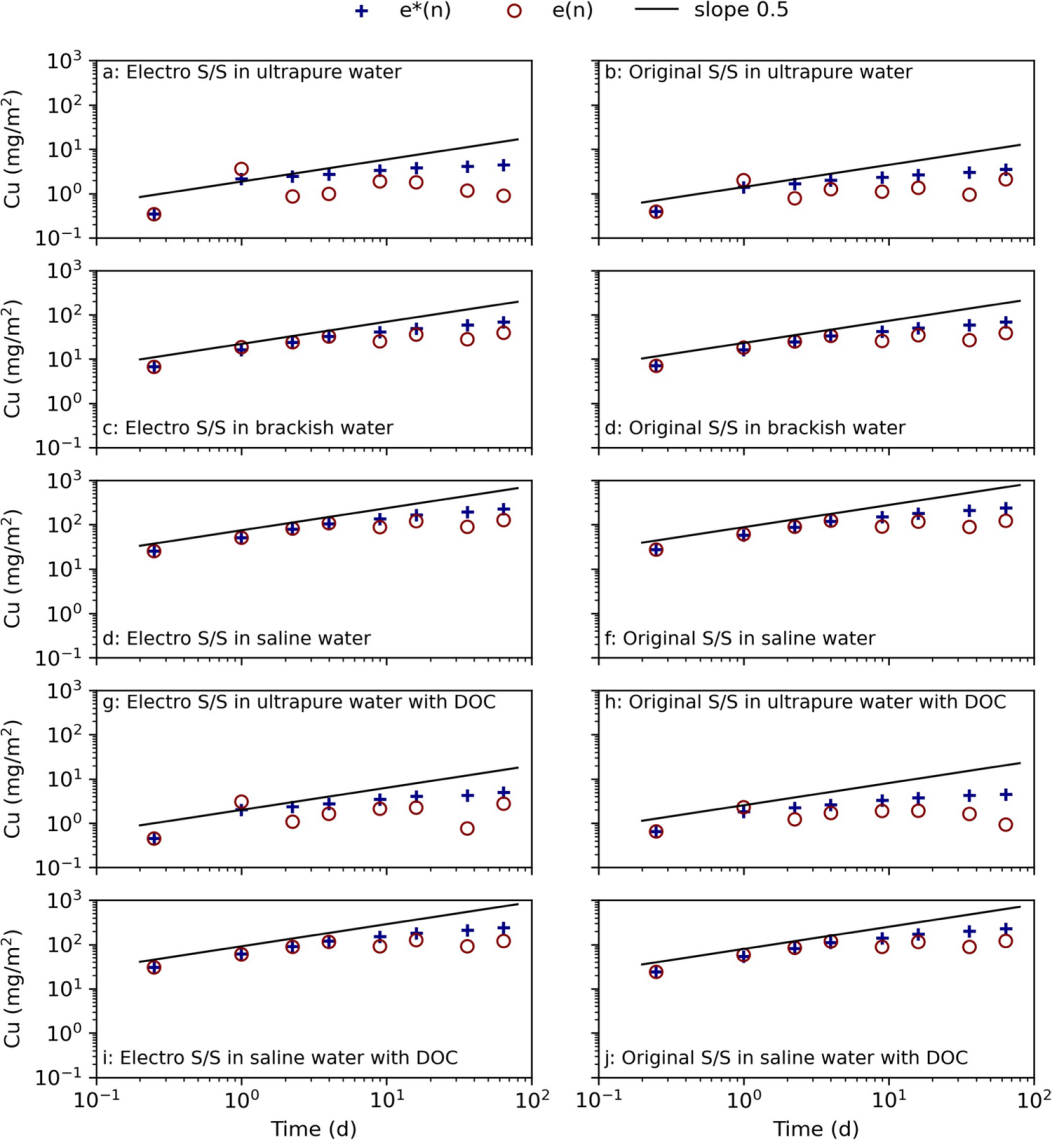
Cu leaching from electrochemically pretreated (electro) and untreated (original) stabilized samples cured in ultra-pure water, brackish water, and saline water, as well as ultra-pure water with dissolved organic carbon (DOC) and saline water with DOC in the NEN7375 surface diffusion leaching test. The curve e*(n) displays the measured cumulative leaching, and *e*(n) displays the derived cumulative leaching. The slope 0.5 is marked as a reference for identifying diffusion-controlled leaching (Environment Agency (2005))

The metals that had a content above the sediment background levels according to the Norwegian classification were and could pose an environmental risk were Cd, Cu, and Zn (Table 1) (Direktoratsgruppen vanndirektivet 2018). The results for Cd were omitted as the measured average concentrations were too low to be assessed according to the NEN7375 test standard. The low release indicates that Cd was efficiently immobilized, probably due to the high pH (Wang, et al. 2021). The electro stabilized samples had a lower Zn release in comparison to the original stabilized samples, probably because electrochemical treatment had lowered the Zn content in the sediment (Table 1). The surrounding saline conditions favored a higher release of Zn and Cu than in ultra-pure water (Fig. 5 and Fig. 6). Both these metals were identified to have low leaching in deionized water, mainly due to the high pH and since the metals have a low solubility (Barjoveanu, et al. 2018). The addition of DOC to the ultra-pure water seems to have resulted in a lowered release of Zn from the electro stabilized sample (Fig. 5).

The leaching of Cu in ultra-pure water from electro stabilized sediment depleted after 9 days, while the leaching from the original stabilized sediment in ultra-pure water continued throughout the test time (Fig. 6). The same trend in the leaching pattern was also seen for Mg (Fig. 4) but not for Zn (Fig. 5). The indication of depletion, seen by the downwards going leaching curves, was also seen in ultra-pure water with DOC for both electro and original stabilized samples (Fig. 6). It was seen that the leaching agents containing DOC resulted in a slightly higher release of Cu, probably due to Cu’s affiliation to binding to organic compounds (Kalmykova, et al. 2008). The presence of DOC had a limited effect on the leaching of most of the other metals, and the leached-out concentrations were in general at levels or slightly higher than the corresponding sample without added DOC as seen for, e.g., Cu. Instead, most metals had a lower release in ultra-pure water compared to in saline water, which has also been seen in previous studies (e.g., Barjoveanu, et al. (2018), Han et al. (2019), Schmukat et al. (2012)). The higher metal release in ultra-pure water may be due to the low ionic strength of the liquid, forcing metal ions to be leached out. This implies that the salinity is more important for the leaching of metals than the presence of DOC (Han, et al. 2019).

#### Leaching according to SS-EN 12457–4

##### Organotin compounds

Organotin compounds were analyzed in the leachates from the 90-day-old stabilized samples that have been cured in ultra-pure water or saline water, crushed, and leached in saline water. Analysis of DBT and MBT concentrations in the leachates was all below the detection limit (< 10 ng/L). In Table 2, it is seen that the TBT content in the stabilized Original S/S 90 NaCl sample was almost three times higher than in the other samples. Despite a higher TBT content, the sample did not release more TBT in comparison to the other stabilized samples (Table 3). The low difference in leached TBT quantity despite the large difference in TBT content in stabilized samples indicate that S/S is an effective method to prevent TBT from being leached out. The sample that released the least TBT was Electro S/S 90 NaCl (228 ng/kg DW) (Table 3). The pretreatment seems to have lowered and in combination with saline curing conditions, hindered the release of TBT. The saline curing environment also had the lowest amount of TBT released from stabilized samples in the NEN7375 leaching tests and also yielded the highest compression strength (Fig. 2). This indicates that both the solidification (increasing the structural integrity) effects and stabilization of TBT (reducing the release of contaminants) were best in saline conditions. These results indicate that the standard procedure for curing using water with low ionic strength could misjudge the performance of the S/S sediment in field conditions (Drincic, et al. 2017).

##### Metals

**Fig. 7:**
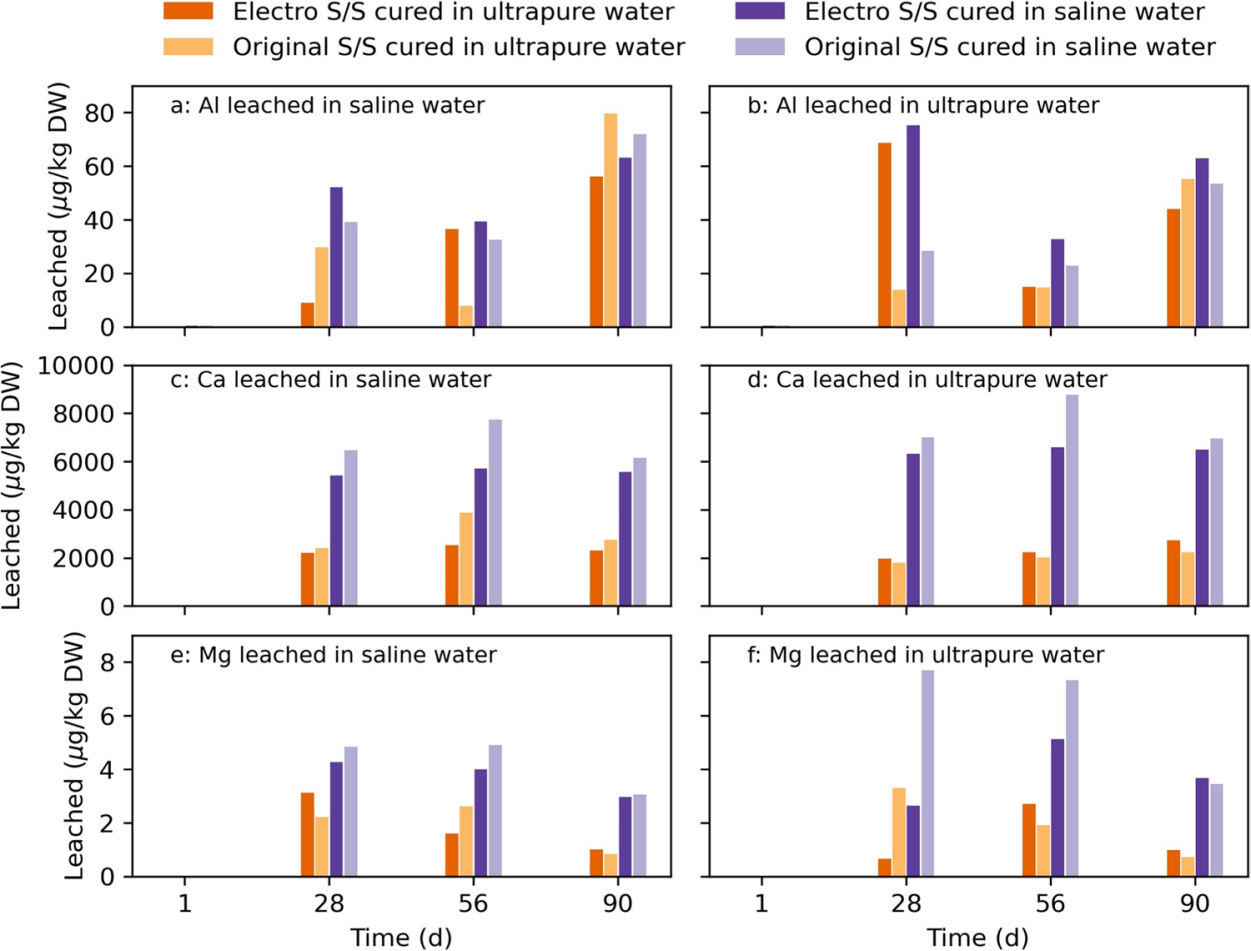
Metals released (µg/kg DW) during the granular waste compliance L/S10 leaching test, leached in either ultra-pure water or saline water. Each uniquely colored bar is corresponding to either untreated (original) or electrochemically pretreated (electro) stabilized samples cured in ultra-pure water or saline water. The number on the horizontal axis denotes the number of days the samples have been curing when the leaching test was conducted

Stabilized samples that had been cured for 1, 28, 56, and 90 days in ultra-pure or saline water were crushed and leached in ultra-pure or saline water solutions. Fig. 1 In this L/S10 leaching tests, the lowest amount of metals was released from the freshly 1-day-old stabilized sediments (Fig. 7 and Appendix Fig. A.6). At this early time in the S/S process, many reactions occur in the stabilized sample, potentially temporarily reducing the amount of metals available for leaching. For samples that had been cured for ≥ 28 days, the leaching seems to be less time dependent. The lack of a significant change in the metal release for the samples during the investigated time interval could imply that the stabilizing effect does not improve after day 28. An ionic equilibrium may have been reached for some components during the curing once the hydrolysis reactions subside, while in a natural environment (similar to the designated site), water movements would probably buffer the high pH and affect the ionic transfer, e.g., through dilution. This could potentially result in a constant low leaching, as seen from leaching experiments using slag (Han, et al. 2019) and in the NEN7375 test in which continuous leaching was observed for most samples. However, in a modified NEN7375 test, it was seen that more metals leached out when the water was not substituted (Drincic, et al. 2017). This is similar to the conditions during the curing of the test pieces, which could indicate that fewer metals are available for the L/S10 leaching as leaching also occurs during the curing

The curing environment was more important than pretreatment for the release of metals in the L/S10 leaching test (Fig. 7 and Fig. A.6). All samples cured in ultra-pure water displayed a lower metal release compared to samples cured in saline water, regardless of the salinity of the leaching agent. This is likely due to the L/S10 leachates’ high pH (~ 11.5), which decreases the mobility of most metals. The high pH of the stabilized sediment be buffered by the surrounding water in situ and a higher metal release could be expected. These results could further imply that the stabilizing effect is better for metals when curing in ultra-pure water in comparison to saline water, as this was also seen in the NEN7375 leaching test. The difference between curing in standardized laboratory ultra-pure water and more realistic saline water containing dissolved ions shows the importance of investigating leaching behavior in the environment to which the stabilized sediment will be exposed (Han, et al. 2019; Schmukat, et al. 2012). This is important for both environmental regulators and constructors to be aware of, as criteria for strength and leaching might need to be changed based on the field conditions.

The leaching of Ca did not differ much regardless of the leaching agents’ salinity (Fig. 7). However, the stabilized samples leached almost double the amount of Mg in saline water in comparison to ultra-pure water. In general, the original stabilized samples leached more Mg compared to the electro stabilized samples, which could indicate that Mg is bonded in Mg-S–H in the electro stabilized samples, as earlier discussed in for metals in the NEN7375 test (Xing, et al. 2009). For Al, the leaching from the 28-day-old electro stabilized samples deviates from all other result (Fig. 7). The increased leaching of Al on day 28 could indicate dissolution of hydrates which could affect the strength of the original S/S as earlier discussed (Marchon and Flatt 2016; Zhang, et al. 2020). However, in the NEN7375 leaching test, the Al leaching curves appeared similar for all samples (Fig. A.4). This indicates that more Al is available within samples than what is leached out through surface diffusion and that the L/S10 test could be useful to identify chemical changes inside the matrix and could capture a time-specific view of the stabilization processes inside the specimens.

## Future perspectives

In this study, electrochemical treatment reduced TBT and metal content in the sediment, and most electrolyzed stabilized samples had a lower or similar metal release in comparison to the original stabilized samples in the L/S10 leaching tests, see Fig. A.6. Optimization of the method would be needed to treat larger amounts of sediments in full-scale applications (e.g., electrode size, current applied). In addition, the method could be optimized to achieve a higher degradation of contaminants and metal recovery. To further reduce the metal leaching, other treatment steps could be added to the electrolysis procedure. Another option may be to investigate different techniques to further reduce the contaminant content and recover metals before stabilization is done (e.g., sediment washing and density separation to remove TBT which often is caught in paint flakes) (Norén, et al. 2021; Turner 2021). This study also shows that changes in the sediment caused by the electrochemical treatment need to be further investigated, together with how such changes affect the S/S reactions, and thereby the strength and leaching behavior.

It should also be noted that the stabilization recipe used was specifically developed for the original sediment from the sampling site and modifications to the recipe can be investigated to better suit pretreated sediment and achieve improved strength properties. Figure 2 shows that the strength has decreased for the electrochemically treated stabilized sediment cured in ultra-pure water after 90 days. Longer testing time could be done to investigate the further strength development over time. It is possible that once most reactions stop, the strength will reach a constant level.

Additionally, in a cold climate such as in Sweden, freeze–thaw cycles can affect the strength and leachability of the stabilized sediment, as the water within the stabilized sample expands during freezing and can cause micro-cracks (Makusa, et al. 2016). It could therefore be of interest to see how freeze–thaw cycles would influence the strength and leaching properties of electrochemically pretreated stabilized sediment. The stabilized sediments would during in situ conditions be surrounded by water that is continuously exchanged, so tests including curing and leaching with continuous replenishment of water would better mimic the in situ conditions.

## Conclusions

The increasing demand for alternatives for sediment disposal makes S/S a technique that is gaining increasing interest as contaminated sediment could be used as a construction material. However, despite the method’s stabilizing effect on contaminants, TBT and metals still leach out from constructions. This study is the first to investigate if electrochemical pretreatment of sediment would lower the leachability and improve strength development after stabilization. Both original (untreated) and electrolyzed stabilized sediment passed the set compression strength limit of 140 kPa for the stabilization recipe used. The best solidification effect was seen for the original (i.e., untreated) stabilized sediment where the highest compression strength was reached.

Regardless of whether electrochemical pretreatment has been used or not, a saline curing environment was effective to increase the strength development. However, a saline curing environment was also identified to enhance the leaching of metals from the stabilized sediment. This highlights the importance of investigating the leaching and strength development for site-specific criteria as there is a risk that the strength and leaching will differ between field and laboratory settings. Electrochemical pretreatment reduces the sediment’s TBT and metal content, in particular for Zn (44% reduction). The release of Zn was lower from electrolyzed stabilized samples than from the original stabilized samples, but the treatment had a low effect on preventing the release of other contaminants after stabilization. If the electrolysis is further optimized (e.g., using larger electrodes), more metals could potentially be removed from the sediment, and the recovered metals could potentially be reused in society. However, sediment electrolysis on a larger scale needs to be further developed to ensure efficient TBT degradation. Also, a better understanding of what occurs and changes within the sediment during the electrolysis treatment and how the treatment impacts the stabilization and solidification reactions is a must to optimize the treatment and outcome for the stabilized sediment. Potentially, another pretreatment or additional treatment steps may be more suitable before stabilization to increase the strength or decrease leaching from the stabilized sediment. Alterations in the S/S recipe may also be required to further lower the leaching and increase the strength.

### Supplementary Information

Below is the link to the electronic supplementary material.

Supplementary file1 (PDF 1.89 MB)

## Data Availability

The data supporting the results reported in this paper can be accessed by contact with the authors.
