# Supplementary material for: The effects of electrochemical pretreatment and curing environment on strength and leaching of stabilized/solidified contaminated sediment
Source: Environ Sci Pollut Res Int. 2023 Dec 22;31(4):5866–80. doi: 10.1007/s11356-023-31477-6 (PMC10799133; doi:10.1007/s11356-023-31477-6)
Supplement: Supplementary file 1 — Supplementary file1 (PDF 1.89 MB) [file 11356_2023_31477_MOESM1_ESM.pdf]

Appendix for
The effects of electrochemical pretreatment and curing environment on strength and leaching of stabilized/solidified contaminated sediment

Anna Norén[a,*], Ann-Margret Strömvall[a], Sebastien Rauch[a], Yvonne Andersson-Sköld[c,d], Oskar Modin[a], Karin Karlfeldt Fedje[a,b]

Addresses

[a] Department of Architecture and Civil Engineering, Division of Water Environment Technology, Chalmers University of Technology, SE-412 96 Gothenburg, Sweden

[b] Recovery and Management, Renova AB, Box 156, SE-401 22 Gothenburg, Sweden

[c] Swedish National Road and Transport Research Institute (VTI), Box 8072, SE-402 78 Gothenburg, Sweden

[d] Department of Architecture and Civil Engineering, Division of Geology and geotechnics, Chalmers University of Technology, SE-412 96 Gothenburg, Sweden

** Corresponding author, anna.noren@chalmers.se*

Table A.1 Typical chemical composition range of Ecocem GGBS (Ecocem, n.d.)

Compound	Composition range (%)
**SiO_2**	34.15-37.9
**Al_2O_3**	10.4-13.1
**Fe_2O_3**	0.64-0.7
**CaO**	37.6-43
**MgO**	7-8.1
**MnO**	0.4
**TiO_2**	0.5-0.7
**SO**	0.01-0.20
**Cl**	0.01-0.03
**S^{2-}**	0.7-1.02
**Na_2O_{eqv}**	0.51-0.53

Table A.2 Average content of compounds in Byggcement Std PK Skövde (Cementa, 2021)

Compound	Composition (%)
**CaO**	61.2
**SiO_2**	18.2
**Al_2O_3**	5
**Fe_2O_3**	2.9
**MgO**	1.2
**Na_2O**	0.16
**K_2O**	1.2
**SO_3**	3.6
**Cl**	0.03

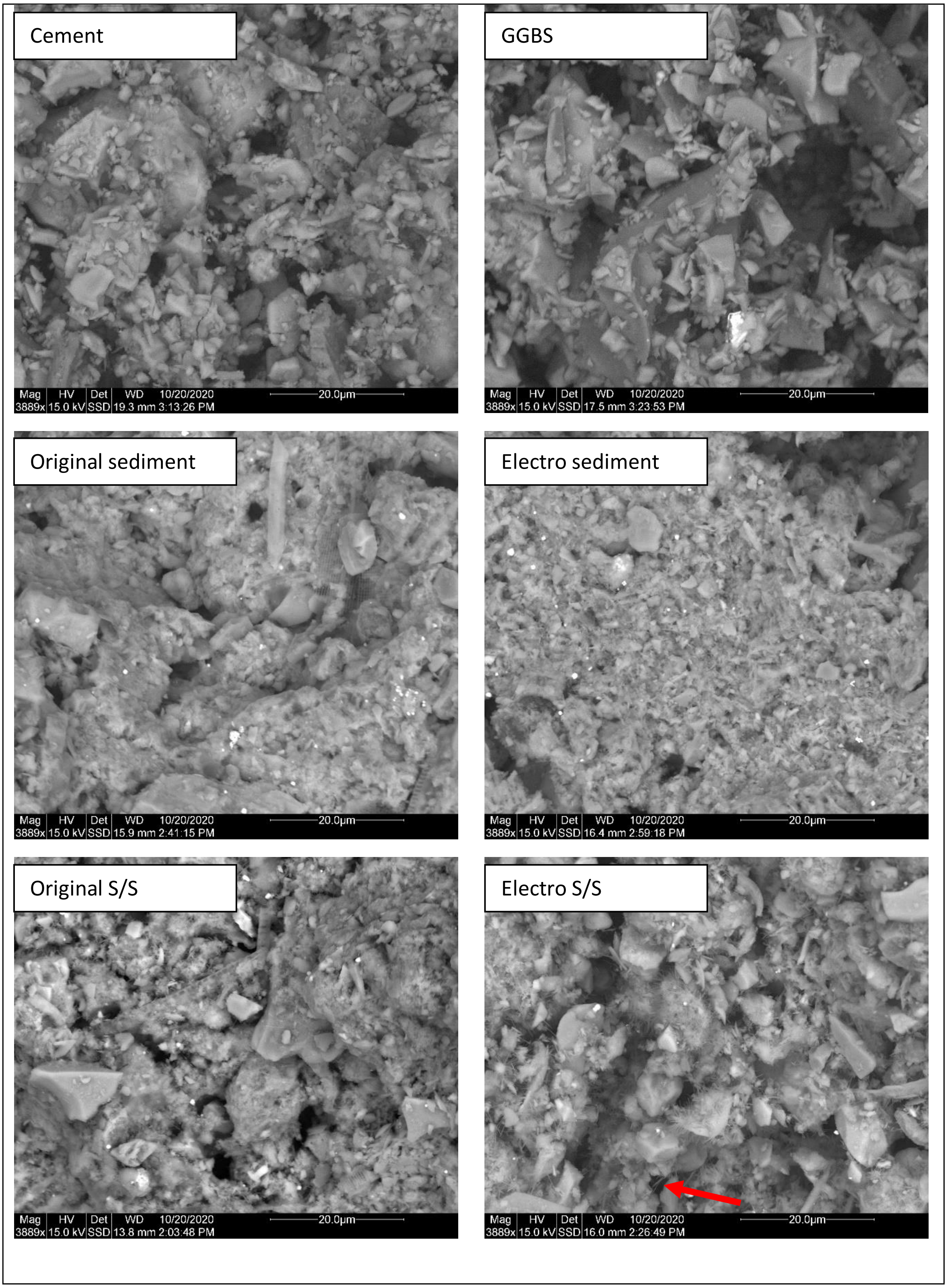
Cement
Mag HV Det WD 10/20/2020 20.0µm
3889x 15.0 kV SSD 19.3 mm 3:13:26 PM
GGBS
Mag HV Det WD 10/20/2020 20.0µm
3889x 15.0 kV SSD 17.5 mm 3:23:53 PM
Original sediment
Mag HV Det WD 10/20/2020 20.0µm
3889x 15.0 kV SSD 15.9 mm 2:41:15 PM
Electro sediment
Mag HV Det WD 10/20/2020 20.0µm
3889x 15.0 kV SSD 16.4 mm 2:59:18 PM
Original S/S
Mag HV Det WD 10/20/2020 20.0µm
3889x 15.0 kV SSD 13.8 mm 2:03:48 PM
Electro S/S
Mag HV Det WD 10/20/2020 20.0µm
3889x 15.0 kV SSD 16.0 mm 2:26:49 PM

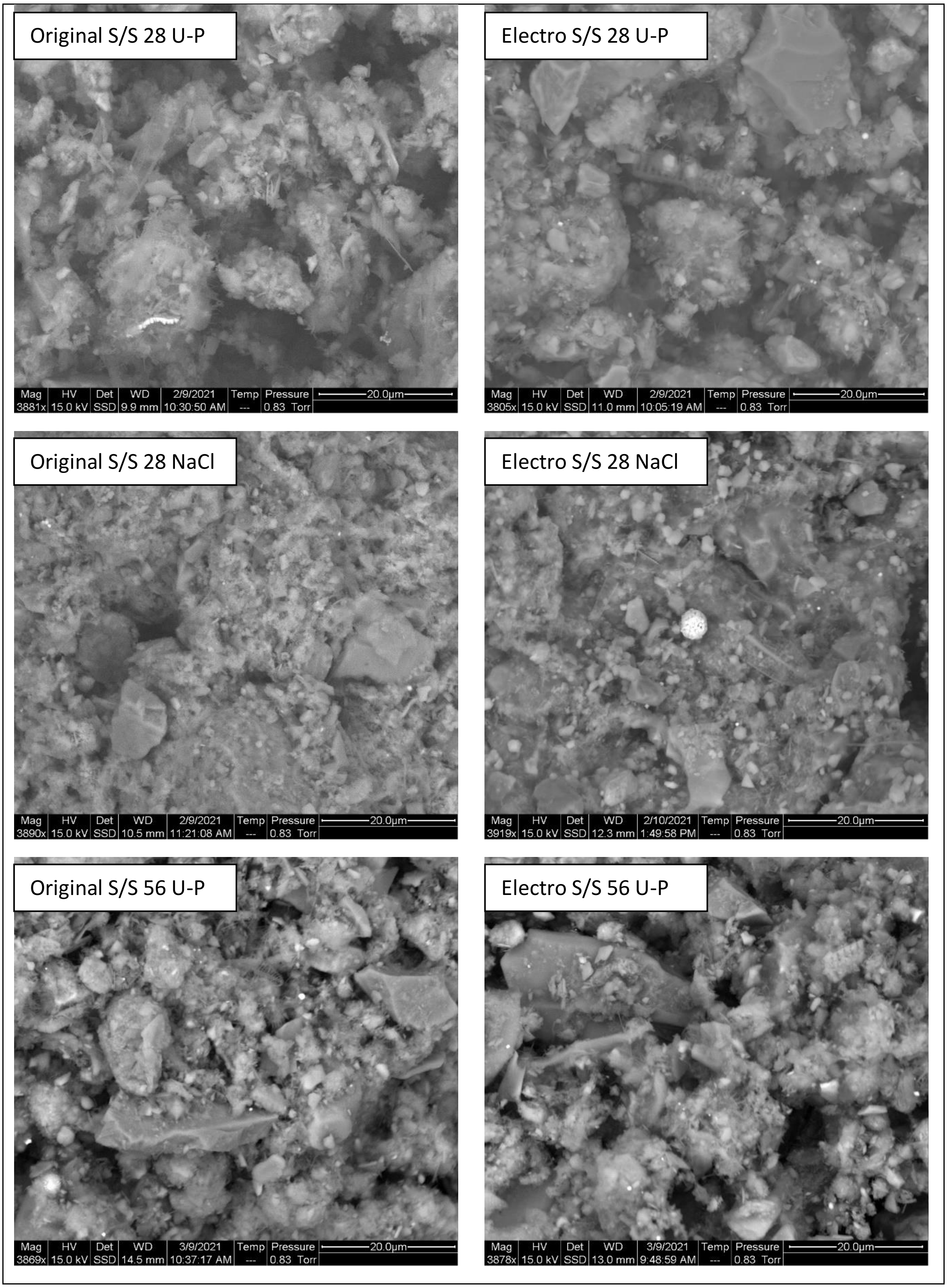
Original S/S 28 U-P
Mag HV Det WD 2/9/2021 Temp Pressure 20.0μm
3881x 15.0 kV SSD 9.9 mm 10:30:50 AM --- 0.83 Torr
Electro S/S 28 U-P
Mag HV Det WD 2/9/2021 Temp Pressure 20.0μm
3805x 15.0 kV SSD 11.0 mm 10:05:19 AM --- 0.83 Torr
Original S/S 28 NaCl
Mag HV Det WD 2/9/2021 Temp Pressure 20.0μm
3890x 15.0 kV SSD 10.5 mm 11:21:08 AM --- 0.83 Torr
Electro S/S 28 NaCl
Mag HV Det WD 2/10/2021 Temp Pressure 20.0μm
3919x 15.0 kV SSD 12.3 mm 1:49:58 PM --- 0.83 Torr
Original S/S 56 U-P
Mag HV Det WD 3/9/2021 Temp Pressure 20.0μm
3869x 15.0 kV SSD 14.5 mm 10:37:17 AM --- 0.83 Torr
Electro S/S 56 U-P
Mag HV Det WD 3/9/2021 Temp Pressure 20.0μm
3878x 15.0 kV SSD 13.0 mm 9:48:59 AM --- 0.83 Torr

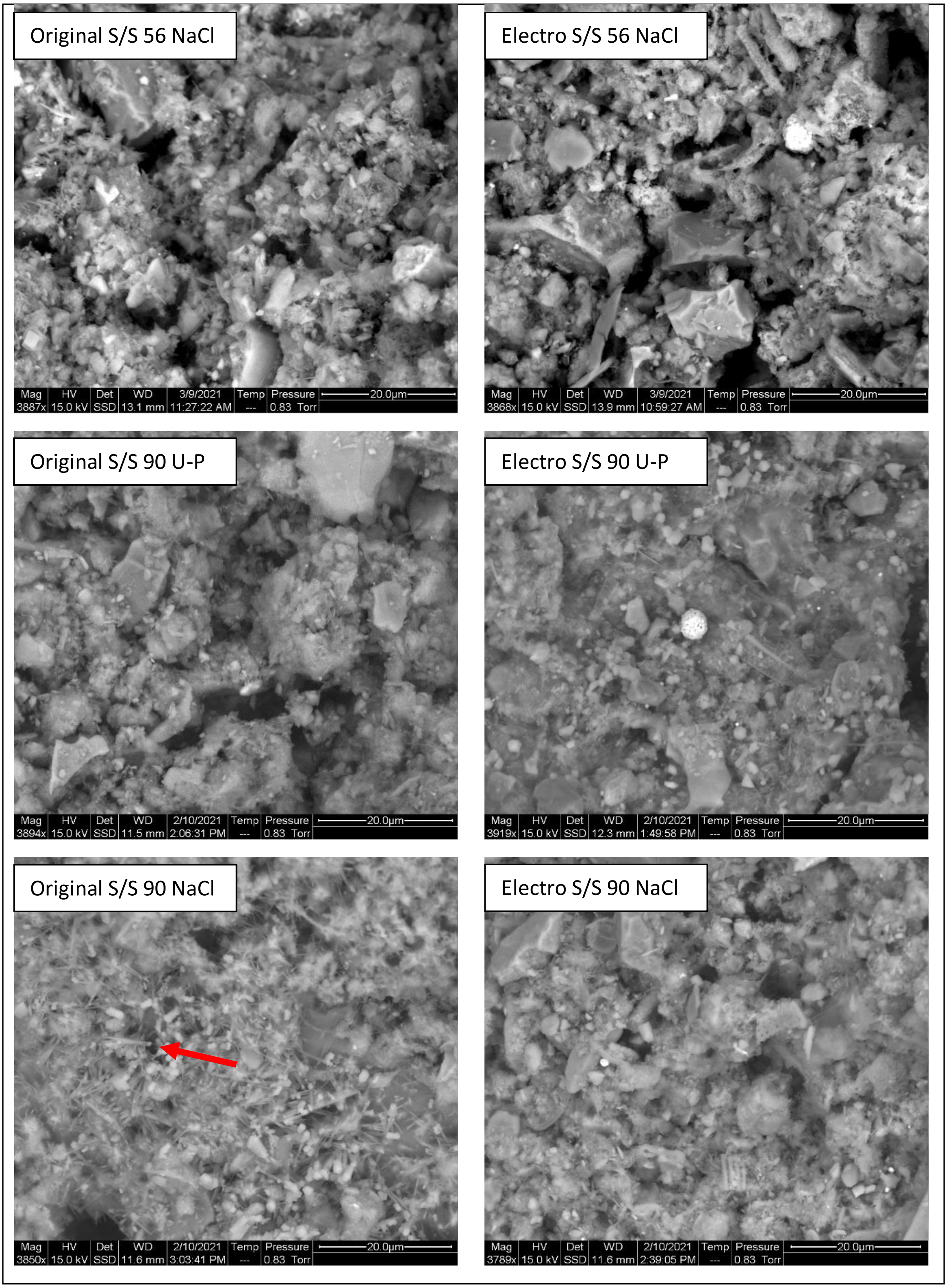

Fig. A.1 ESEM images of cement, ground granulated blast-furnace slag (GGBS), untreated (original) sediment, electrochemically pretreated (electro) sediment, stabilized original samples (original S/S), and stabilized electrochemically pretreated sediment (electro S/S). The number in the stabilized samples' image name identifies the time in days after the casting. The curing conditions for the stabilized sediments were either in ultra-pure (U-P) or saline (NaCl) water. In samples Electro S/S and Original S/S 90 NaCl red arrows point out an example of the long crystals seen in the figure (presumingly ettringite).

Table A.3 Results from the compression tests for electrochemically pretreated stabilized sediment (electro S/S) and untreated stabilized sediment (original S/S) cured in ultra-pure (U-P) or saline (NaCl) water

Day	Electro S/S U-P kPa	Original S/S U-P kPa	Electro S/S NaCl kPa	Original S/S NaCl kPa
**28**	1343	444	973	630
**28**	873	965	1280	800
**28**	1160	913	1342	751
**56**	1295	1643	1215	1696
**56**	1411	1681	1232	1693
**56**	1355	1712	1243	1720
**56**	1042	1677	-	-
**90**	442	2282	1359	2557
**90**	345	2153	1308	2485
**90**	226	2012	1280	2514
**90**	351	2436	-	-

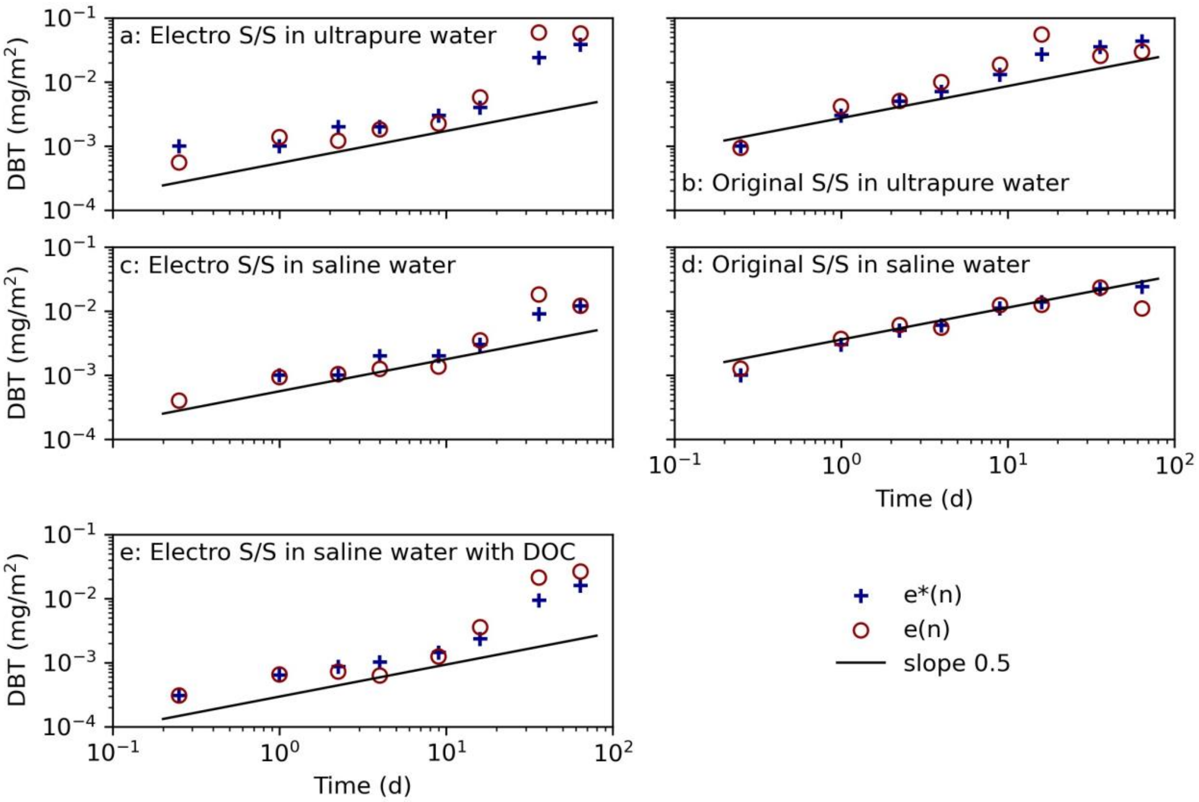

Fig. A.2 DBT leaching from untreated (i.e., original) and electrochemically pretreated (i.e., electro) stabilized samples (S/S) cured in ultra-pure water, saline water, as well as saline water with dissolved organic carbon (DOC) in the NEN7375 surface diffusion leaching test. The curve e*(n) displays the measured cumulative leaching and e(n) displays the derived cumulative leaching. The slope 0.5 is marked as a reference for identifying diffusion-controlled leaching (Environment Agency, 2005)

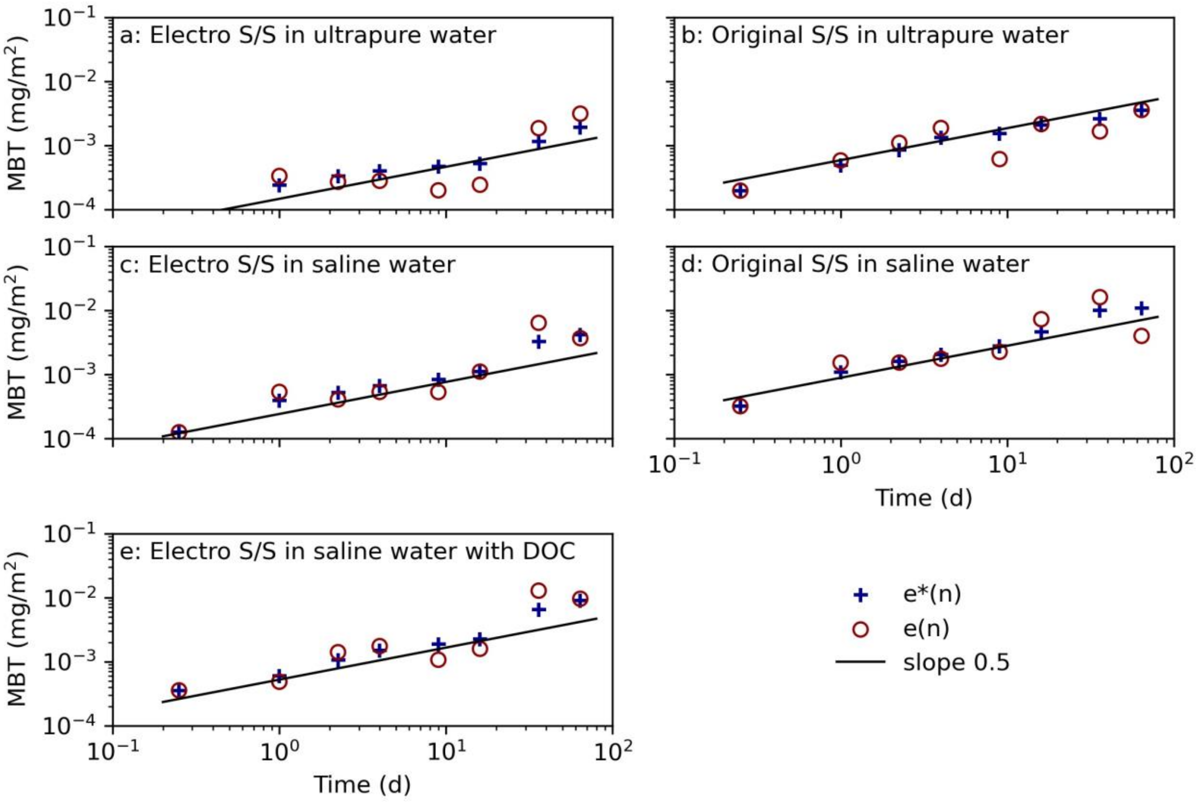

Fig. A.3 MBT leaching from untreated (i.e., original) and electrochemically pretreated (i.e., electro) stabilized samples (S/S) cured in ultra-pure water, saline water, as well as saline water with dissolved organic carbon (DOC) in the NEN7375 surface diffusion leaching test. The curve e*(n) displays the measured cumulative leaching and e(n) displays the derived cumulative leaching. The slope 0.5 is marked as a reference for identifying diffusion controlled leaching (Environment Agency, 2005)

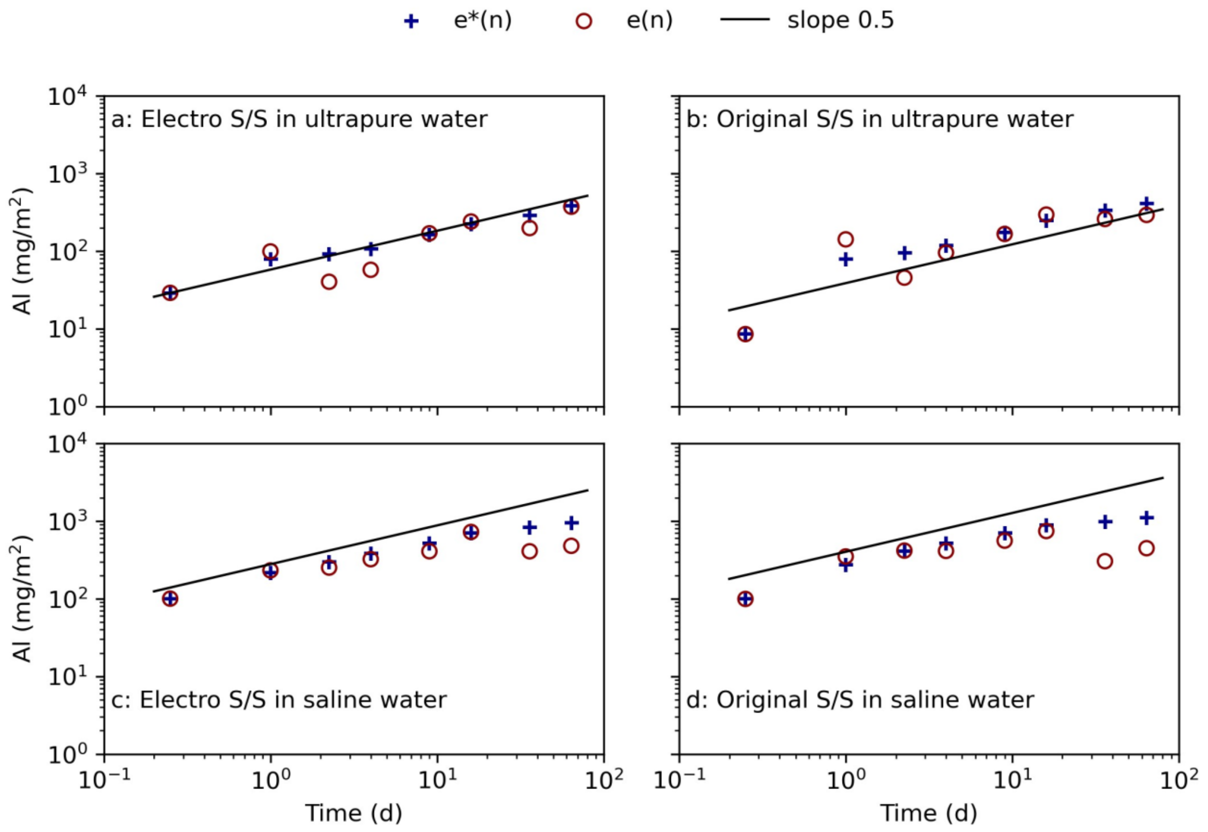

Fig. A.4 Al leaching from untreated (original) and electrochemically pretreated (electro) stabilized samples (S/S) cured in ultra-pure water and saline water in the NEN7375 surface diffusion leaching test. The curve e*(n) displays the measured cumulative leaching and e(n) displays the derived cumulative leaching. The slope 0.5 is marked as a reference for identifying diffusion-controlled leaching (Environment Agency, 2005)

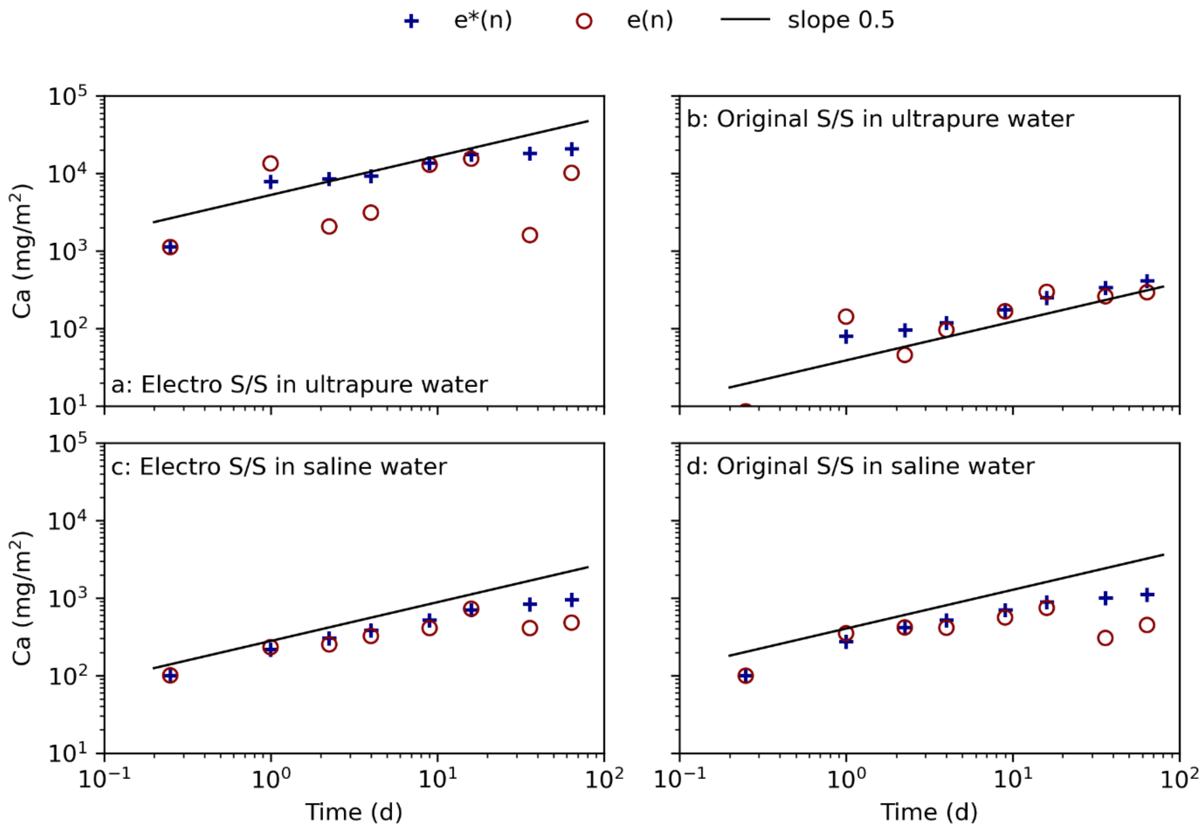

Fig. A.5 Ca leaching from untreated (original) and electrochemically pretreated (electro) stabilized samples (S/S) cured in ultra-pure water and saline water in the NEN7375 surface diffusion leaching test. The curve e*(n) displays the measured cumulative leaching and e(n) displays the derived cumulative leaching. The slope 0.5 is marked as a reference for identifying diffusion-controlled leaching (Environment Agency, 2005)

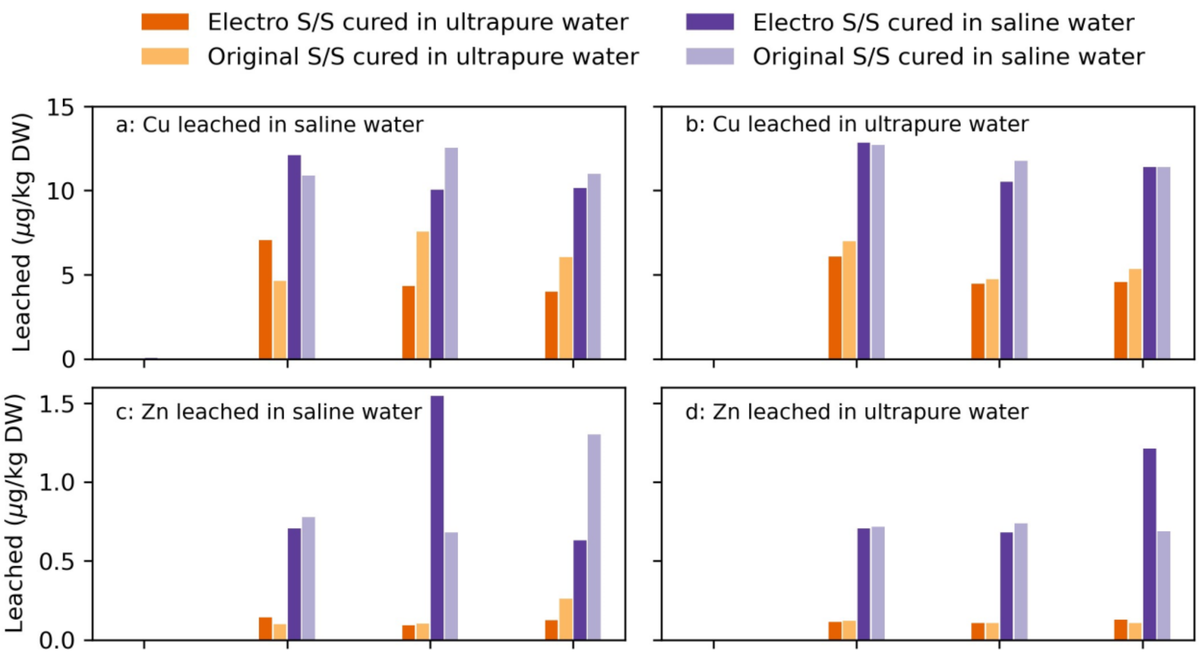

Fig. A.6 Metals released (µg/kg DW) during the granular waste compliance L/S10 leaching test, leached in either ultra-pure water or saline water. Each uniquely colored bar is corresponding to either untreated (i.e., original) or electrochemically pretreated (electro) stabilized samples cured in ultra-pure water or saline water. The number on the horizontal axis denotes the number of days the samples have been curing when the leaching test was conducted

References

Cementa, 2021. Typanalys 2020 (in Swedish)

Ecocem, n.d. GGBS - Technical data sheet.

Environment Agency, 2005. EA NEN 7375:2004 Leaching characteristics of moulded or monolitic building and waste material. Determination of leaching of inorganic components with diffusion test. "The tank test". Version 1.0 April 2005
